# Bis(morpholino)methan: MAK-Begründung, Nachtrag

**DOI:** 10.34865/mb562590kskd10_1ad

**Published:** 2025-03-31

**Authors:** Andrea Hartwig, MAK Commission

**Affiliations:** 1 Institut für Angewandte Biowissenschaften. Abteilung Lebensmittelchemie und Toxikologie. Karlsruher Institut für Technologie (KIT) Adenauerring 20a, Geb. 50.41 76131 Karlsruhe Deutschland; 2 Ständige Senatskommission zur Prüfung gesundheitsschädlicher Arbeitsstoffe. Deutsche Forschungsgemeinschaft, Kennedyallee 40, 53175 Bonn, Deutschland. Weitere Informationen: Ständige Senatskommission zur Prüfung gesundheitsschädlicher Arbeitsstoffe | DFG

**Keywords:** Bis(morpholino)methan, Nase, oberer Atemtrakt, Reizwirkung, Kanzerogenität, Formaldehydabspalter, Strukturanalogie, Keimzellmutagenität

## Abstract

The German Senate Commission for the Investigation of Health Hazards of Chemical Compounds in the Work Area (MAK Commission) has re-evaluated bismorpholinomethane [5625-90-1] with regard to its carcinogenicity and germ cell mutagenicity classification, its ability to be absorbed through the skin, its sensitization potential and the derivation of an occupational exposure limit value (maximum concentration at the workplace, MAK value) can be derived. Relevant studies were identified from a literature search and also unpublished study reports were used. Bismorpholinomethane is corrosive to the skin of rabbits. The substance is a formaldehyde releaser and is expected to undergo rapid hydrolysis in aqueous solution. The local irritation is therefore attributed to the hydrolysis products formaldehyde and morpholine. The carcinogenicity, toxicity and genotoxicity induced by bismorpholinomethane in the upper respiratory tract or nose, the likely target organs, have not been investigated. The substance exhibited mutagenic and clastogenic potential in vitro, presumably due to the release of formaldehyde. Formaldehyde was classified in Carcinogen Category 4 because it induces tumours in nasal tissues at concentrations that exceed their detoxification capacity. As a formaldehyde releaser, bismorpholinomethane could likewise be classified in Carcinogen Category 4. However, because it is not possible to derive a MAK value for bismorpholinomethane, the substance has been assigned to Carcinogen Category 2 with the footnote “Prerequisite for Category 4 in principle fulfilled, but insufficient data available for the establishment of a MAK or BAT value”. As there are no data for the systemic bioavailability of bismorpholinomethane and the formaldehyde that is released in tissues by hydrolysis, there is no experimental evidence that the formaldehyde reaches the germ cells. Therefore, bismorpholinomethane has been classified in Category 3 B for germ cell mutagens. Clinical data in humans reveal a skin sensitizing potential that is also caused by the release of formaldehyde. Bismorpholinomethane has been designated with the “Sh” notation. Skin contact is not expected to contribute significantly to systemic toxicity.

**Table:** 

**MAK-Wert**	**–**
**Spitzenbegrenzung**	**–**

**Hautresorption**	**–**
**Sensibilisierende Wirkung (2017)**	**Sh**
**Krebserzeugende Wirkung (2024)**	**Kategorie 2^a)^**
**Fruchtschädigende Wirkung**	**–**
**Keimzellmutagene Wirkung (2024)**	**Kategorie 3 B**

**BAT-Wert**	**–**

CAS-Nr.	5625-90-1
Formel	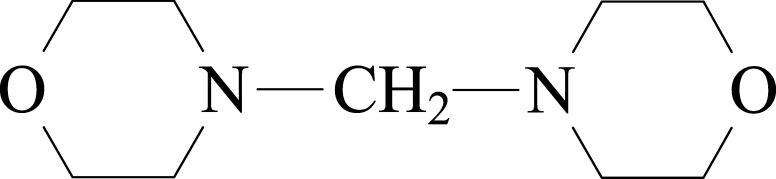
	C_9_H_18_N_2_O_2_
Molmasse	186,25 g/mol
Dampfdruck	0,00625 hPa (ber. für 25 °C mit EpiSuite 3.1.2); 0,0043 hPa (ber. für 20 °C mit EUSES) (UBA GmbH 2014)
pH-Wert	10,8 (Hartwig und MAK Commission 2018)
**1 ml/m^3^ (ppm) ≙ 7,728 mg/m^3^**	**1 mg/m^3^ ≙ 0,129 ml/m^3^ (ppm)**

^a)^ Voraussetzung für Kategorie 4 prinzipiell erfüllt, aber Daten für MAK- oder BAT-Wert-Ableitung nicht ausreichend

Hinweis: Formaldehydabspalter. Der Stoff kann gleichzeitig als Dampf und Aerosol vorliegen.

Es liegen eine Begründung (Greim 1999) und ein Nachtrag zu allen Endpunkten (Hartwig und MAK Commission 2018) vor. Bis(morpholino)methan ist bisher dem Abschnitt II b der MAK- und BAT-Werte-Liste zugeordnet. 

Dieser Nachtrag wurde erstellt, da eine weitere Hydrolyse-Studie zur Verfügung steht. Es erfolgte eine Neubewertung der Daten zur Formaldehydabspaltung nach aktuellem Vorgehen der Kommission (DFG 2024). Unter diesem Aspekt wurden in diesem Nachtrag die Hydrolyse sowie die Endpunkte Kanzerogenität, Keimzellmutagenität und MAK-Wert neu bewertet. Zudem wurden die Endpunkte Hautresorption und Sensibilisierung geprüft. Zitierte unveröffentlichte Studien von Firmen wurden der Kommission zur Verfügung gestellt. 

## Allgemeiner Wirkungscharakter

Bis(morpholino)methan ist ein Formaldehydabspalter und hydrolysiert schnell in wässriger Lösung. Der vermutlich kritische Effekt von Bis(morpholino)methan ist die kanzerogene und reizende Wirkung am Atemtrakt. Bis(morpholino)methan ist in vitro mutagen an Bakterien sowie mutagen und klastogen an Säugetierzellen. Die Klastogenität in vitro hat sich in vivo nicht bestätigt. In-vivo-Untersuchungen zur mutagenen Wirkung liegen nicht vor. Zur Kanzerogenität gibt es zwei Studien mit subkutaner Gabe an Ratten, in denen Tumoren an der Injektionsstelle beobachtet worden sind. Der Stoff ist hautsensibilisierend beim Menschen. Ein Bericht weist auf eine atemwegssensibilisierende Wirkung von Bis(morpholino)methan hin.

## Toxikokinetik und Metabolismus

### Hydrolyse

Bis(morpholino)methan hydrolysiert in wässriger Lösung, wobei ein Molekül Formaldehyd und zwei Moleküle Morpholin entstehen (Greim 1999). Daneben bildet sich N-Methylolmorpholin (Hartwig und MAK Commission 2018).

Die Hydrolysegeschwindigkeit von Bis(morpholino)methan ist abhängig von der Konzentration der Lösung, dem pH-Wert und der Temperatur. Mit der Erniedrigung des pH-Wertes, der Erhöhung der Temperatur oder zunehmender Verdünnung nahm auch das Ausmaß der Hydrolyse zu (Daten nicht gezeigt) (BASF 2007; Hartwig und MAK Commission 2018). 

Bei 2,4-stündiger Inkubation von 0,5 g Bis(morpholino)methan/l bei 37 °C (pH 1,2) und bei 50 °C (pH 4, 7 und 9) lag die gemessene Konzentration von Bis(morpholino)methan zu allen Zeitpunkten (k. w. A.) unterhalb der Nachweisgrenze von 3,18 mg/l. Nach Methode C.7 in Richtlinie 92/69/EWG wurde die Halbwertszeit bei 25 °C daher auf deutlich weniger als einen Tag geschätzt (UBA GmbH 2014). 

Bei 60 °C hydrolysierten Bis(morpholino)methan-Lösungen (0,15 % bis 100 %, pH-Werte 2 bis 11) so schnell, dass sofort nach dem Ansetzen keine Ausgangssubstanz mehr gemessen werden konnte. Eine Hydrolyse-Geschwindigkeit konnte daher nicht bestimmt werden (BASF 2007).

Eine In-vitro-Studie zur dermalen Aufnahme und eine Studie mit intratrachealer Instillation (Hartwig und MAK Commission 2018) belegen qualitativ, dass Bis(morpholino)methan bei Kontakt mit biologischem Material zu Formaldehyd und Morpholin hydrolysiert. Hierbei verschiebt sich das Gleichgewicht der Hydrolyse von Bis(morpholino)methan durch Verdünnung und durch die Reaktion von Formaldehyd mit biologischem Material schnell zu Formaldehyd und Morpholin (UBA GmbH 2014).

**Fazit:** Aufgrund der schnellen Hydrolyse und des Fehlens genauer Daten wird als Worst-Case-Abschätzung davon ausgegangen, dass der Abbau des aus Bis(morpholino)methan entstehenden Formaldehyds im Atemtrakt langsamer erfolgt als dessen Bildung. 

## Erfahrungen beim Menschen

### Allergene Wirkung

#### Kontaktallergene Wirkung

Die bereits im letzten Nachtrag (Hartwig und MAK Commission 2018) aufgeführten klinischen Beobachtungen im Zusammenhang mit Kühlschmierstoffen zeigen ein hautsensibilisierendes Potenzial von Bis(morpholino)methan, das sicherlich auf die Freisetzung von Formaldehyd zurückgeführt werden kann. 

Von insgesamt 1497 Personen, die im Testzeitraum von 2007–2020 wegen beruflich bedingter Kontaktdermatitis in einer Klinik untersucht wurden, wurden 369 Personen mit Bis(morpholino)methan (1 % in Vaseline) getestet. Davon reagierten 22 (6,0 %) positiv, wobei 20 (91 %) dieser Personen gleichzeitig auf Formaldehyd reagierten. Die Ablesung erfolgte bis mindestens zum 4. Tag, wobei die einzelnen Ablesetage sowie die Reaktionsstärken nicht berichtet sind (Aalto-Korte und Pesonen 2021).

#### Atemwegssensibilisierende Wirkung.

Im letzten Nachtrag (Hartwig und MAK Commission 2018) wurde bereits ein Bericht zur immunologischen Wirkung des Bis(morpholino)methan an den Atemwegen beschrieben, der auf eine atemwegssensibilisierende Wirkung von Bis(morpholino)methan hinweist (Walters et al. 2013).

Seit dem letzten Nachtrag liegen keine neuen Daten zur atemwegssensibilisierenden Wirkung vor. 

## Tierexperimentelle Befunde und In-vitro-Untersuchungen

### Allergene Wirkung

Seit dem letzten Nachtrag liegen keine neuen Daten vor. Die darin beschriebenen Studien wurden mit zu geringen Konzentrationen durchgeführt, sodass die negativen Ergebnisse nicht zur Bewertung herangezogen werden (Hartwig und MAK Commission 2018).

### Genotoxizität

Bis(morpholino)methan wirkt in vitro mutagen an Bakterien sowie mutagen und klastogen an Säugetierzellen. Die in vitro gezeigte Klastogenität hat sich nach oraler Gabe weder in einem Mikronukleustest an Mäusen noch einem UDS-Test an Ratten bestätigt. In-vivo-Untersuchungen zur mutagenen Wirkung liegen nicht vor. Ähnlich wie bei Formaldehyd könnten in Abhängigkeit von der freigesetzten Formaldehydmenge lokal genotoxische Effekte auftreten (Hartwig und MAK Commission 2018). Neue Daten zur genotoxischen Wirkung liegen nicht vor.

Auch Formaldehyd ist nach oraler Gabe negativ im Maus-Mikronukleustest, aber positiv mit hohen intraperitonealen Dosen (Greim 2000), sodass aufgrund des negativen oralen In-vivo-Tests eine keimzellmutagene Wirkung von Bis(morpholino)methan nach Inhalation nicht völlig ausgeschlossen werden kann.

### Kanzerogenität

In zwei Studien mit subkutaner Gabe traten bei Ratten Tumoren an der Injektionsstelle auf (Greim 1999; Hartwig und MAK Commission 2018). Neue Daten zur Kanzerogenität liegen nicht vor.

## Bewertung

Kritische Effekte sind die kanzerogene und lokal reizende Wirkung des Hydrolyseprodukts Formaldehyd sowie die sensibilisierende Wirkung an der Haut.

**Krebserzeugende Wirkung. **Es liegen keine Kanzerogenitäts-Langzeitstudien mit Bis(morpholino)methan vor. Bis(morpholino)methan selbst weist nur in vitro ein genotoxisches Potenzial auf, eine mögliche genotoxische Wirkung am wahrscheinlichen Zielgewebe des oberen Atemtrakts bzw. der Nase (wie bei Formaldehyd) ist jedoch nicht untersucht.

Die lokale Kanzerogenität des Hydrolyseprodukts Formaldehyd hingegen ist ausführlich dokumentiert (Greim 2000; Hartwig 2010). Formaldehyd ist in Kanzerogenitäts-Kategorie 4 eingestuft, da es erst bei Konzentrationen, die die Entgiftungskapazitäten des Nasengewebes überschreiten, in diesem Gewebe kanzerogen wirkt. Bis(morpholino)methan spaltet Formaldehyd schnell ab (siehe Abschnitt „Hydrolyse“). Untersuchungen, wie viel Formaldehyd im Atemtrakt freigesetzt wird, liegen nicht vor. Es fehlt die Bestimmung der Halbwertszeit in der Nase unter physiologischen Bedingungen, also bei 37 °C und pH-Wert um etwa 7. Daher wird für die Bewertung am Arbeitsplatz bei inhalativer und dermaler Exposition von einer sofortigen vollständigen Formaldehydfreisetzung ausgegangen. Aufgrund der lokalen kanzerogenen Wirkung von Formaldehyd könnte Bis(morpholino)methan in Analogie zu Formaldehyd in Kanzerogenitäts-Kategorie 4 eingestuft werden. Da jedoch kein MAK-Wert für Bis(morpholino)methan abgeleitet werden kann, wird der Stoff der Kanzerogenitäts-Kategorie 2 zugeordnet und erhält die Fußnote „Voraussetzung für Kategorie 4 prinzipiell erfüllt, aber Daten für MAK- oder BAT-Wert-Ableitung nicht ausreichend“.

Bei Anwesenheit von nitrosierenden Agenzien ist unter Umständen mit dem Auftreten von Nitrosomorpholin, einem kanzerogenen N-Nitrosamin, zu rechnen (Greim 1999). 

**MAK-Wert. **Es liegen keine Inhalationsstudien mit Bis(morpholino)methan am Menschen oder am Tier vor, aus denen ein MAK-Wert abgeleitet werden kann. 

Bis(morpholino)methan wirkt ätzend an der Kaninchenhaut. Der Stoff hydrolysiert beim Kontakt mit biologischem Material, wobei aus einem Molekül Bis(morpholino)methan ein Molekül Formaldehyd und zwei Moleküle Morpholin entstehen können (Hartwig und MAK Commission 2018) und eine inhalationstoxische Wirkung durch Formaldehyd und Morpholin zu erwarten ist.

Morpholin wirkt ätzend an der Kaninchenhaut und am Auge und verursacht in einer chronischen Inhalationsstudie an Ratten ab 50 ml/m^3^ Nekrosen in den Nasenturbinaten (MAK-Wert 10 ml/m^3^ (36 mg/m^3^)) (Greim 1996). Der Dampfdruck von Morpholin beträgt 9,8 hPa bei 20,3 °C (ECHA 2022). Formaldehyd hat einen MAK-Wert von 0,3 ml/m^3^ (0,37 mg/m^3^), der vor der lokalen kanzerogenen Wirkung schützt und aus der augenreizenden Wirkung abgeleitet ist (Greim 2000; Hartwig 2010). Der Dampfdruck von Formaldehyd beträgt bei 25 °C 5185 hPa (OECD 2002).

Da der MAK-Wert von Morpholin wesentlich höher ist als der von Formaldehyd, ist nur dessen Wirkung für die Bewertung von Bis(morpholino)methan relevant. Der Dampfdruck von Bis(morpholino)methan (0,004 bis 0,006 hPa; ber. für 20 bzw. 25 °C) zeigt, dass der Stoff bei Konzentrationen, bei denen Formaldehyd schon dampfförmig ist, noch als Aerosol vorliegen kann. 

Für Aerosole ist durch die Impaktierung im Atemtrakt mit einer stärkeren Wirkung als die durch dampfförmigen Formaldehyd verursachte zu rechnen, wie mit einem anderen Formaldehydabspalter, zu dem eine Inhalationsstudie vorliegt, gezeigt wurde (siehe Begründung N,N′,N′′-Tris(-hydroxyethyl)hexahydro-1,3,5-triazin; Hartwig und MAK Commission 2023). Ein MAK-Wert in Analogie zu Formaldehyd kann daher nicht abgeleitet werden. Da auch keine NOAEC aus einer Inhalationsstudie vorliegt, kann für Bis(morpholino)methan kein MAK-Wert festgesetzt werden. Eine Spitzenbegrenzung entfällt.

Bei Anwendung in verdünnten wässrigen Lösungen sollte mit einer vollständigen Hydrolyse gerechnet und daher der MAK-Wert für Formaldehyd (Greim 2000; Hartwig 2010) eingehalten werden.

**Keimzellmutagene Wirkung. **Bis(morpholino)methan wirkt in vitro mutagen an Bakterien sowie mutagen und klastogen an Säugetierzellen. Die Klastogenität in vitro hat sich in vivo nicht bestätigt. In-vivo-Untersuchungen zur mutagenen Wirkung liegen nicht vor (Hartwig und MAK Commission 2018). 

Formaldehyd ist in Kategorie 5 für Keimzellmutagene eingestuft. Dies bedeutet, dass bei Einhaltung des MAK-Wertes von 0,3 ml/m^3^ nur ein sehr geringer Beitrag zum genetischen Risiko für den Menschen zu erwarten ist (Greim 2000; RAC und SEAC 2020).

Bis(morpholino)methan könnte zwar in Analogie zu Formaldehyd in die Kategorie 5 für Keimzellmutagene eingestuft werden, jedoch kann für Bis(morpholino)methan kein MAK-Wert festgesetzt werden. 

Da Daten zur systemischen Bioverfügbarkeit von Bis(morpholino)methan und dem durch Hydrolyse freigesetzten Formaldehyd fehlen, liegt kein experimenteller Beleg vor, dass freigesetzter Formaldehyd in aktiver Form die Keimzellen erreicht. Wegen des Verdachts auf eine genotoxische Wirkung wird Bis(morpholino)methan in Kategorie 3 B für Keimzellmutagene eingestuft.

**Fruchtschädigende Wirkung. **Es liegen keine Daten zur fruchtschädigenden Wirkung vor. Da kein MAK-Wert aufgestellt werden kann, entfällt die Zuordnung zu einer risikobasierten Schwangerschaftsgruppe. In einer pränatalen Entwicklungstoxizitätsstudie nach OECD-Prüfrichtlinie 414 an Kaninchen traten bis zur höchsten getesteten Dosis von 100 mg Bis(morpholino)methan/kg KG und Tag keine entwicklungstoxischen Effekte auf (Hartwig und MAK Commission 2018). Aus den vorliegenden Daten lässt sich kein Verdacht auf eine entwicklungstoxische Wirkung ableiten.

**Hautresorption. **Daten zur transdermalen Aufnahme beim Menschen liegen für Bis(morpholino)methan nicht vor. In einer In-vitro-Studie an Humanhaut nach OECD-Prüfrichtlinie 428 wurde ^14^C-markiertes Bis(morpholino)methan in einer nicht-ätzenden Konzentration von 3 % eingesetzt. Die Gesamtaufnahme nach 24 Stunden betrug 68 %, woraus ein transdermaler Flux von 8,5 µg/cm^2^ und Stunde abgeleitet wurde. Für eine Exposition unter Standardbedingungen von 2000 cm^2^ Hautfläche und einer Dauer von einer Stunde ergibt sich daraus eine Gesamtaufnahme von 17 mg. Demgegenüber wurde eine systemisch tolerable Menge von 1530 mg Bis(morpholino)methan aus den Ergebnissen einer 90-tägigen Schlundsondenstudie an Ratten sowie einer toxikokinetischen Übertragung auf den Menschen errechnet, so dass keine „H“-Markierung vergeben wurde (Hartwig und MAK Commission 2018).

Unter der Annahme einer raschen Hydrolyse mit Bildung von einem Molekül Formaldehyd pro Molekül Bis(morpholino)methan lässt sich die Zunahme des Formaldehydspiegels im Blut nach dermaler Applikation einer nicht ätzenden 3%igen wässrigen Lösung von Bis(morpholino)methan unter Standardbedingungen abschätzen: aus den zuvor berechneten insgesamt 17 mg Bis(morpholino)methan (0,09 mmol) werden 2,74 mg Formaldehyd über einen Zeitraum von einer Stunde freigesetzt, pro Minute demnach etwa 46 µg bzw. 57 µg/1,25 Minuten (mittlere Halbwertszeit des Formaldehyds im Blut; Kaden et al. 2010). Die transdermale Aufnahme von Bis(morpholino)methan in den letzten sechs Halbwertszeit-Intervallen führt demnach zu einer im Blut zirkulierenden Formaldehydmenge von (57 + 29 + 15 + 8 + 4 + 2) µg = 115 µg bzw. etwa 0,12 mg. 

Der physiologisch bedingte Formaldehydspiegel im Blut des Menschen beträgt etwa 2–3 mg/l (10–15 mg in 5 l Blut, frei und reversibel gebunden) (Heck et al. 1985). Der zusätzliche Eintrag durch transdermal aufgenommenes Bis(morpholino)methan erhöht demnach die Gleichgewichtskonzentration von Formaldehyd im Blut nicht bedeutend, sodass der Stoff weiterhin nicht mit „H“ markiert wird.

**Sensibilisierende Wirkung. **Auch eine neue klinische Studie bestätigt das hautsensibilisierende Potenzial von Bis(morpholino)methan beim Menschen, das sicherlich auf die Freisetzung von Formaldehyd zurückgeführt werden kann. Verwertbare tierexperimentelle Untersuchungen liegen weiterhin nicht vor. Insgesamt wird Bis(morpholino)methan daher weiterhin mit „Sh“ markiert. Zur immunologischen Wirkung des Bis(morpholino)methan an den Atemwegen liegt lediglich ein Bericht vor, der bereits in der letzten Begründung aufgeführt ist und auf eine atemwegssensibilisierende Wirkung von Bis(morpholino)methan hinweist. Seitdem liegen jedoch keine neuen Daten vor, sodass gemäß den Kriterien der Kommission Bis(morpholino)methan weiterhin nicht mit „Sa“ markiert wird.

## References

[ref_5F7JLL5R] Aalto-Korte Kristiina, Pesonen Maria (2021). Patterns of positive patch test reactions to formaldehyde and formaldehyde releasers at the Finnish Institute of Occupational Health from 2007 to 2020.. Contact Dermatitis.

[ref_N8HDW4HJ] BASF (2007). Hydrolysestudie bei verschiedenen pH-Werten, Konzentrationen und Temperaturen.

[ref_UR27D8ZV] Deutsche Forschungsgemeinschaft DFG (2024). MAK- und BAT-Werte-Liste 2024. Maximale Arbeitsplatzkonzentrationen und Beurteilungswerte in biologischem Material. Ständige Senatskommission zur Prüfung gesundheitsschädlicher Arbeitsstoffe, Mitteilung 60. Erratum.

[ref_SN2XLVG2] (2022). Morpholine (CAS Number 110-91-8). Registration dossier. Joint submission, first publication 02 Mar 2011, last modification 06 Apr 2022.

[ref_GGLJJ7GR] Greim H (1996).

[ref_ZLN7N6JP] Greim H (1999).

[ref_4PHW3NZM] Greim H (2000).

[ref_JSIFQZXU] Hartwig A, Hartwig A (2010).

[ref_SZPTP64L] Hartwig A., MAK Commission (2018). Bis(morpholino)methan. MAK Value Documentation in German language. MAK Collect Occup Health Saf.

[ref_9Y2HXDMU] Hartwig A., MAK Commission (2023). N,N′,N′′-Tris(β-hydroxyethyl)hexahydro-1,3,5-triazin. MAK-Begründung, Nachtrag. MAK Collect Occup Health Saf.

[ref_VE7479IY] Heck H, Casanova-Schmitz M, Dodd P.B, Schachter E.N, Witek T.J, Tosun T (1985). Formaldehyde (CH_2_O) concentrations in the blood of humans and Fischer-344 rats exposed to CH_2_O under controlled conditions.. Am Ind Hyg Assoc J.

[ref_CH7LPTVP] Kaden D.A., Mandin C, Nielsen G.D., Wolkoff P, World Health Organisation WHO (2010). WHO guidelines for indoor air quality: selected pollutants.

[ref_W8LPG2J8] Organisation for Economic Co-operation and Development OECD (2002). Formaldehyde (CAS No 50-00-0). OECD SIDS Initial Assessment Report.

[ref_2VIQPMTI] Committee for Risk Assessment RAC, Committee for Socio-economic Analysis SEAC (2020). Opinion on an Annex XV dossier proposing restrictions on formaldehyde and formaldehyde releasers. ECHA/RAC/RES-O-0000006740-76-01/F, RAC opinion, adopted 13 March 2020.

[ref_WRM7Z5CU] Umweltbundesamt GmbH UBA GmbH (2014). CLH report – Proposal for harmonised classification and labelling – based on regulation (EC) No 1272/2008 (CLP Regulation), Annex VI, Part 2. Substance name: 4-(morpholin-4-ylmethyl)morpholine, EC Number: 227-062-3, CAS Number: 5625-90-1. Version number: 2, 15 September 2014.

[ref_ZAXHEM22] Walters Gareth I., Moore Vicky C., Robertson Alastair S., McGrath Emmet E., Parkes Edward, Burge P. Sherwood (2013). Occupational asthma from sensitisation to 4,4-methylene-bismorpholine in clean metalworking fluid. Eur Respir J.

